# The role of economic evaluation in the decision-making process of family physicians: design and methods of a qualitative embedded multiple-case study

**DOI:** 10.1186/1471-2296-10-15

**Published:** 2009-02-11

**Authors:** Chantale Lessard, André-Pierre Contandriopoulos, Marie-Dominique Beaulieu

**Affiliations:** 1 Department of Health Administration, Faculty of Medicine, University of Montreal, Montreal, Quebec, Canada; 2 Department of Family Medicine, Doctor Sadok Besrour Chair in Family Medicine, Faculty of Medicine, University of Montreal, Montreal, Quebec, Canada

## Abstract

**Background:**

A considerable amount of resource allocation decisions take place daily at the point of the clinical encounter; especially in primary care, where 80 percent of health problems are managed. Ignoring economic evaluation evidence in individual clinical decision-making may have a broad impact on the efficiency of health services. To date, almost all studies on the use of economic evaluation in decision-making used a quantitative approach, and few investigated decision-making at the clinical level. An important question is whether economic evaluations affect clinical practice. The project is an intervention research study designed to understand the role of economic evaluation in the decision-making process of family physicians (FPs). The contributions of the project will be from the perspective of Pierre Bourdieu's sociological theory.

**Methods/design:**

A qualitative research strategy is proposed. We will conduct an embedded multiple-case study design. Ten case studies will be performed. The FPs will be the unit of analysis. The sampling strategies will be directed towards theoretical generalization. The 10 selected cases will be intended to reflect a diversity of FPs. There will be two embedded units of analysis: FPs (micro-level of analysis) and field of family medicine (macro-level of analysis). The division of the determinants of practice/behaviour into two groups, corresponding to the macro-structural level and the micro-individual level, is the basis for Bourdieu's mode of analysis. The sources of data collection for the micro-level analysis will be 10 life history interviews with FPs, documents and observational evidence. The sources of data collection for the macro-level analysis will be documents and 9 open-ended, focused interviews with key informants from medical associations and academic institutions. The analytic induction approach to data analysis will be used. A list of codes will be generated based on both the original framework and new themes introduced by the participants. We will conduct within-case and cross-case analyses of the data.

**Discussion:**

The question of the role of economic evaluation in FPs' decision-making is of great interest to scientists, health care practitioners, managers and policy-makers, as well as to consultants, industry, and society. It is believed that the proposed research approach will make an original contribution to the development of knowledge, both empirical and theoretical.

## Background

Health economics is the branch of economics concerned with how scarce health care resources are allocated to maximise the health of the community [1,2]. Economic evaluations use analytic techniques to assess the relative costs and consequences of health care technologies [2,3]. By "technology" we mean any health care intervention, program or service, including, among other things: devices; drugs; instruments; genetic screening; equipment and facilities; genomics; medical and surgical procedures; professional practices; rehabilitation; alternative medicine; methods of organizing services; and vaccination. The role of economic evaluation is to provide rigorous data to inform and improve the health care decision-making process [1-3]. It is clear that in Canada evolution of the health care system under pressure of policies for cost-containment is creating a growing consciousness of the importance of resource allocation [4,5]. The issues of technology assessment and economic evaluation are given special attention in the final report of the Commission on the Future of Health Care in Canada [5]. It remains unclear if this will result in a more rational demand for economic evaluations. The process of decision-making takes place at different levels of the health care system: macro (policy), meso (administrative) and micro (clinical practice). Since planning, managing and providing care do not entail the same imperatives [6], the decision-makers' attitudes towards economic evaluations as an aid for decision-making may also differ [7,8]. The micro level covers the resource allocation decisions made by individual health care professionals at a patient level [7,9]. It is at that particular level that most decision-making occurs, and thus, where economic evaluation evidence should have the most extensive influence [10]. An important question is whether economic evaluations affect clinical practice. Since every decision has an opportunity cost, ignoring economic evidence in individual clinical decision-making may have a broad impact on the efficiency of health services [11].

Primary health care is one of the key priorities in the Action Plan agreed to by governments across Canada for renewing the health care system [12,13]. Strong primary care may improve health outcomes, increase cost-effectiveness, and promote social equity [14]. This means that the family physician or general practitioner (FP) is expected to take care of the individual patient's need as well as taking into account common resource use [15-19]. This will depend on the quality and validity of the knowledge influencing the decision-making process. As FPs deal with individual patients on a case-by-case basis, it is highly important, then, to ensure they have access to useful and high-quality information on the economic consequences of health technologies [4,5,20]. Primary care FPs may be isolated from the scientific world [21]. These clinicians may be influenced by brief reading, but in particular by their many informal interactions with peers and opinion leaders, and with pharmaceutical representatives and other sources of largely tacit knowledge [21,22]. One of the most important challenges facing the world of research today is to develop effective knowledge translation strategies specific to the primary care sector, where 80% of all health problems are treated [21].

Findings from health services research consistently show a gap between evidence and practice [23,24]. Professional and social networks play a major role in the types of evidence being used and in determining the characteristics of practice contexts [25-28]. It is now recognized that the practice of medicine is influenced by many factors other than evidence, although none is sufficient alone to explain clinical decision-making [16,29,30]. Clinical decision-making can be considered as a complex social process with multiple factors, mediated by individual and social contexts [31-34]. Ethical considerations have also become part of the decision-making process [35]. Some physicians have been invoking intuition in confronting the challenges of daily clinical practice [36]. At the same time, physicians are now expected to respect patients' autonomy in clinical decision-making [15]. Finally, some argue that there may be a paradox between the need for evidence-based medicine (EBM) and the unique predicament, context, preferences and choices of the individual patient [37].

The interest in economic evaluation in health care has increased considerably since the early 1990s [3,38]. However, investigations have shown that the influence of economic evaluation on decision-making and the knowledge about the formal methodology are rather limited [7,9,39]. A number of barriers to the use of economic evaluation studies in decision-making have been identified, including questions about the reliability, relevance, and timeliness of economic evaluation studies [7,9,39,40]. Despite the aesthetics of economic evaluation models and the precision of computation, decision analyses may oversimplify complex decisions [41,42]. Furthermore, economic evaluation arguments are utilitarian in nature and therefore population-based [43]. Some argue that the current utilitarian approach fails to consider all society's values and health objectives [11,44-47]. In reality, efficiency is often traded off in health services to achieve more equitable allocation of resources [11,48].

### Justification for the research

To date, almost all studies on the use of economic evaluation in health care decision-making used a quantitative approach, and few investigated decision-making at the micro level. Many believe that economic evaluation is mostly an issue for policy-makers and managers. Literature sources have revealed the importance of having decision-makers with an interest in economic evaluation, favourable infrastructures for the use of economic evaluation evidence, and changes made to the way economic evaluations are conducted or presented, if one wishes to influence the level of use [9,49-55]. Although these three conditions seem necessary, we doubt they will be sufficient. We have alternative views.

First, as most health care resource allocation decisions take place at the point of the clinical encounter, we believe that this is where economic evaluation may exert the most extensive influence on decision-making. Second, we believe that lack of influence stems primarily from characteristics of individuals, and social contexts. To pose the question of why economic evaluation findings are not used to directly influence practice overlooks or, at least, subordinates the other factors influencing clinical decision-making [56]. In fact, studies using quantitative methodology tend not to acknowledge the interaction of the individual with the 'outside world' [27,57]. Lastly, we concur with Lomas' [58] comment that decision-making is a complex social process, not a technical task. The reasons why economic evaluation has a role, or not, in the decision-making process of FPs are complex and individual, professional, contextual, environmental and organizational factors are closely entwined. But above all, by understanding that primary care family practices are complex systems, the patterns of relationships between agents and structures may be the strongest factors of influence [59].

Therefore, it is important to explore and understand the social processes influencing FPs' knowledge, perceptions, attitudes, behaviours and views related to the role of economic evaluation evidence in clinical decision-making. Qualitative research may offer a richer and deeper understanding of this complex social phenomenon [60]. There is a need to enhance our understanding of the role of economic evaluation in the decision-making process of FPs from a disciplinary perspective different than health economics [56].

### Purpose and objectives of the research

The project is an intervention research study designed to understand the role of economic evaluation in the decision-making process of FPs. A qualitative case study strategy is proposed. The contributions of the project will be from the perspective of Pierre Bourdieu's sociological theory. Bourdieu's theory of practice provides a powerful framework for understanding the practices of individuals and groups in the social world [61]. His foundational concepts suggest a different conceptualization of the agent, as socially embedded, as embodied dispositions shaped by one's position within social fields [62-64]. As a theoretical research framework, Bourdieu's theory of practice offers the possibility of exploring FPs' relationships and interactions with the structures and agents within the field.

The empirical objectives of the research are to develop a deep understanding of: 1) the social processes that influence FPs' schemes of perception, thought, appreciation and action with respect to the role of economic evaluation evidence in clinical decision-making; 2) the FPs' willingness to contribute to efficient, fair and legitimate resource allocation; and 3) the potential influence of economic evaluation evidence on everyday clinical decision-making.

The theoretical objectives will include: 1) a conceptual model specific to the micro level of decision-making highlighting complex relationships between various agents and structures, and economic evaluations; and 2) a better understanding of the theory and practice of knowledge translation.

### Theoretical framework

#### Bourdieu's theory of practice

French scholar Pierre Bourdieu is one of the most influential social scientists of the twentieth century [65]. Despite his impressive life work and academic influence, Bourdieu has had limited attention in both the health care and health economics literature. Previous uses of Bourdieu's work in health care research include, among other things, analyses and studies of: health-related behaviour [66,67]; psychiatric survivors' voice [68]; sickness absence [69]; and older people's use of medication [70]. It has recently been proposed as a theoretical framework for future nursing research [61].

In his book "The social structures of the economy", Bourdieu [71] is very critical of the intellectualist bias in economics. According to him, economists develop increasingly abstract theories and econometric models, with no concern for reality. By taking for granted assumptions about actors' interests and interactions, economists leave out a very important part of social reality. Because of their socially constructed nature, understanding preferences, behaviours and markets requires serious attention to social reality [71]. The studies of this social scientist have revealed the structuring effects of social fields on their members' beliefs, dispositions, and practices [61,72]. He believed that practices were not the mechanical results of social conditions; neither that individuals were fully-free and independent of social conditions [73,74]. As such, he rejected the idea that practices could simply be explained in terms of individual decision-making [67].

Bourdieu's theory of practice illustrates how the context-sensitive nature of practice must be understood as a socially constituted practical knowledge (i.e., a 'feel for the game') [62,75]. His foundational concepts are habitus, capital, and field. Habitus interacts directly with capital as agents struggle for capital resources but are predisposed by their habitus [61]. However, the habitus and capital are intertwined with a specific field [76]. The influence of the field is crucial, as the action is both informed and constrained by the actual context conditions of the social field an agent is situated in [61,74,76]. Structures and habitus are both *opus operatum *(result of practices) and *modus operandi *(mode of production of practices) [73,74,77]. All three concepts are relational, that is, their definition can only be understood in relation to one another [64].

#### Concept of field

A field is presented as a structured system of social positions, struggle and power relations [61,64,67,78]. "*A field may be defined as a network, or a configuration, of objective relations between positions. These positions are objectively defined, in their existence and in the determinations they impose upon their occupants, agents or institutions, by their present power (or capital) whose possession commands access to the specific profits that are at stake in the field, as well as by their objective relations to other positions (domination, subordination, homology, etc.)*." [64] It is a social arena in which agents and institutions are concerned with access to, control over and struggle for capital [78]. Practices are both informed and constrained by the context of the field [61,74,76].

#### Concept of capital

Bourdieu conceptualizes the resources available to agents in all fields as capital (economic, cultural, social, symbolic), which yields power [61,73,77-79]. "*Capital is accumulated labor (in its materialized form or its 'incorporated', embodied form) which, when appropriated on a private, i.e., exclusive, basis by agents or groups of agents, enables them to appropriate social energy in the form of reified or living labor*." [79] For Bourdieu, the dominant fields are a result of the distribution of the four types of capital. Within each field, there is again a different distribution of the four types of capital and this results in a hierarchy of positions [74].

#### Concept of habitus

For Bourdieu, conditions of existence both generate and shape practices and representations through habitus (systems of dispositions) [61,73,76-78]. "*The conditionings associated with a particular class of conditions of existence produce habitus, systems of durable, transposable dispositions, structured structures predisposed to function as structuring structures, that is, as principles which generate and organise practices and representations that can be objectively adapted to their outcomes without presupposing a conscious aiming at ends or express mastery of the operations necessary in order to attain them. Objectively 'regulated' and 'regular' without being in any way the product of obedience to rules, they can be collectively orchestrated without being the product of the organising action of a conductor*." [77]. The habitus enables agents to construct individual and collective practices. These practices are themselves constitutive of the dispositions of the habitus [76].

### Research questions

1. How does economic evaluation evidence affect FPs' clinical decision-making?

2. What are the FPs' dispositions with respect to the influence of economic evaluation evidence in decision-making? And, how have these dispositions been shaped by their life histories?

3. What is the particular capital at stake in the field of family medicine? And, what are the strategies that FPs use to maintain and improve their social position in the field with respect to the particular capital at stake?

4. How do the wider social relationships in which FPs are involved influence their willingness to contribute to optimal health care resource allocation?

5. How do the concepts of field, capital and habitus contribute to the development of a conceptual framework which enhances our understanding of knowledge translation?

## Methods/design

### Strategic framework

#### Research strategy and researchers' epistemological position

A qualitative research strategy is proposed. With its emphasis on context and holism and due attention to system and situation dynamics, qualitative research may offer a richer and deeper understanding of a complex social phenomenon [60,80-84]. For the purpose of this research, we will adopt a transcendental realism position, which involves the belief that "*social phenomena exist not only in the mind but also in the objective world-and that some lawful and reasonable stable relationships are to be found among them*." [80] Human meanings and intentions are worked out within structures, institutions, practices and conventions that create regularities and patterns [80]. According to Miles and Huberman [80], the transcendental realist approach to qualitative research incorporates an interpretive element: "*We agree with interpretivists who point out that knowledge is a social and historical product and that 'facts' come to us laden with theory. We affirm the existence and importance of the subjective, the phenomenological, the meaning-making at the center of social life. Our aim is to register and 'transcend' these processes by building theories to account for a real world that is both bounded and perceptually laden, and to test these theories in our various disciplines*."

#### Research design

We propose to conduct an embedded multiple-case study design. Case study is an appropriate research design to document a contemporary phenomenon within its real-life context, particularly when the boundaries between phenomenon and context are not clearly evident; and in which multiple sources of evidence are used [60,84]. It allows the study of the particularity and the complexity of the case [83]. Furthermore, the case study design is advantageous when "how" and "why" questions are being posed [60]. The multiple-case design that we propose will draw on the following steps developed by Yin [85]: 1) formulating explicit research questions; 2) developing a formal research design; 3) developing hypotheses based on theory and previous research; 4) collecting data to test these hypotheses; 5) creating a case study database (available for inspection by third parties); and 6) conducting qualitative analysis. Our multiple-case design will follow a replication logic. The replication of findings, over multiple cases, may increase confidence in the robustness of the findings [80,85,86]. One or two formative pilot case studies will be conducted to help refine methodological and procedural issues [60,86]. Pilot case report(s) will be written about the lessons learned [60]. Ten case studies will be performed. The FPs will be the unit of analysis. The design will be emergent. This means that facets of the design can be revised or modified as a result of discoveries arising during the course of the research [60,81].

There will be two embedded units of analysis: the FPs (micro-level of analysis) and the field of family medicine (macro-level of analysis). For the micro-level of analysis: It will be important to examine the habitus of FPs as it influences their perceptions, norms, habits and practices. It is the concept of habitus that provides the explanatory link between individual behaviour and social structures [66]. It will be also important to examine the capital they possess as FPs use strategies to maintain or improve their social position in the field with respect to the particular type of capital at stake [61,67,78]. Lastly, it will be important to examine the relationships and interactions between the structures and agents within the field. FPs' perceptions, norms, habits and practices are shaped and reshaped in relation to the perceived individual (physicians, patients) and social contexts (organizational, environmental, professional and political) [87].

For the macro-level of analysis: The concept of habitus always exists in dialectical relationship to the concept of field; in this case, the field of family medicine. This medical professional system has its own history and culture. The family medicine field can be considered to be a separate field but viewed from another angle; it is a subfield within the field of medicine. The field of medicine is itself a subfield within the field of power. It will be important to understand how the specialty of family medicine has evolved (history and culture), and to explore the field of family medicine in relation to the fields of medicine and power, and the discernable forms of capital at stake in the field of family medicine.

#### Quality of the research

Four issues are important for judging the quality of any qualitative work, including case study: objectivity/confirmability, internal validity/credibility, external validity/transferability, and reliability/dependability [80]. The several strategies to be used in dealing with these issues are outlined in Table 1. These strategies will be applied throughout the subsequent conduct of the study [60]. In particular, reflexivity and triangulation will be two of the main strategies to enhance the rigor and the quality. Different types of triangulation will be used, including: methodological triangulation; data source triangulation; investigator/analyst triangulation; and theory/perspective triangulation [82-84,86]. Furthermore, three other approaches to analytical triangulation will also be used: member checking (presenting the researcher's analysis to research participants); audience review (presenting the researcher's analysis to primary intended users of the report); and expert review (presenting the final report (thesis) to doctoral committee and summiting publications and presentations to peer-reviewed scientific journals) [81-83].

**Table 1:** Strategies to improve the quality of the research

Issues*	Strategies**
**Objectivity/confirmability**	Reflexivity/participant objectivation (researcher's biography, values, *a priori *assumptions, perspectives, theoretical biases, etc.) Particularity (doing justice to the integrity of each case) Case study protocol (detailed description of methods and procedures) Project logbook (decisions, procedures, communications, meetings, etc.) Case study database (including case study notes, case study documents, tabular materials, analyses, etc.) Chain of evidence (explicit links between the questions asked, the evidence, and the conclusions drawn) Consideration of competing hypotheses or rival conclusions
**Internal validity/credibility**	Methodological triangulation Data source triangulation Investigator/analyst triangulation Theory/perspective triangulation Specification of the unit of analysis Rich and thick description of context (settings, participants, procedures, etc.) Rigorous and systematic fieldwork procedures Reliability of coding and pattern analyses (using multiple coders) Establish a chain of evidence Integrity in analysis (search for and analysis of alternative themes, divergent patterns, rival explanations and negative cases) Member checking (respondent validation)
**External validity/transferability**	Rich and thick description of context Rich description of findings Keeping methods and data in context (when communicating findings) Use replication logic Audience review (primary intended users of the report) Generation of theoretical statements
**Reliability/dependability**	Strategic design congruent with research questions Paradigm specified Case study protocol, case study database, chain of evidence and project logbook available for review Final coding cross-checked and verified with a second analyst (researcher) Standardized data collection Expert audit review (doctoral committee, peer reviewers for scientific publications and presentations)

*[80]; **[60,80-82,84,86]

### Methodological framework

#### Participants

Qualitative research typically focuses on in-depth understanding on relatively small samples, selected purposefully [80,82]. Our samples will be selected using a combination of purposeful sampling approaches. The sampling strategies will be directed towards theoretical generalization (analytic generalization), and not empirical generalization (statistical generalization) [60,80,81]. The sampling logic will follow the one proposed by Yin [60]: *Each case must be carefully selected so that it either (a) predicts similar results... or (b) predicts contrasting results but for predictable reasons...*

In general, the samples will consist of a minimum of 1 pilot case and 10 case studies. The samples will be selected using three purposeful sampling approaches. First, convenience sampling will be employed for the pilot case(s). The main criteria for selecting the pilot case(s) will be access and geographic proximity [60]. Second, maximum variation (heterogeneity) sampling will be used to select a minimum of eight cases [82]. According to Patton [82], this approach could document the uniqueness of each case, and identify important shared patterns that cut across variations. To keep the research manageable, the cases will consist of FPs in primary care settings and practicing in two areas of the Province of Quebec: Metropolitan Montreal, and Saguenay-Lac-St-Jean. The complexity and uncertainty inherent in medical decision-making are unlikely to vary from one place to another.

A diverse group of FPs will be needed. Based on literature sources, we identified six relevant characteristics for constructing the sample. In addition to the practicing area, diversity will be sought in sex, environmental setting (rural or urban), practice setting (Local Community Services Centre, Family Medicine Group, private group or private solo), training (Quebec Faculties of Medicine: University of Montreal, University of Sherbrooke, University Laval or McGill University) and experience (< 5, 5–15, 16–25 or > 25 years). This means that each of the eight cases will be a unique combination of characteristics, but one sharing some characteristics with one or more cases [80]. Lastly, snowball sampling will be used to identify a minimum of two cases (FPs) who are valuable opinion leaders in Quebec's field of family medicine. The candidate cases will be nominated by a number of key informants and by the eight cases selected with the previous approach.

All candidate cases will be screened beforehand [85]. In particular, the first stage of the maximum variation sampling approach will consist of obtaining archival data from professional associations on the pool of FPs currently practicing in the Province of Quebec. The six relevant characteristics mentioned above will serve as selection criteria. For all sampling approaches, all candidate cases will be solicited by telephone and by mail. Due to time and access constraints, one of the principal selection criteria will be to maximize what can be learned. It will be extremely important to select cases which are not only receptive and willing to participate, but, above all, information-rich [82,83,86]. Information-rich cases are those whose study will offer the opportunity to learn about issues of central importance to the purpose of the research [82]. In the event where a case does not offer potential for learning (for example, because it is not accessible or we cannot spend enough time with it), it shall be dropped and another shall be selected [83].

The 10 selected cases will be intended to reflect a diversity of FPs, and not to be representative. The sampling will be terminated when saturation is reached, i.e., when additional data or information would not contribute to a better understanding of the phenomena in question [82]. This means that one or more cases (pilot, FPs and/or opinion leaders) could be considered.

#### Data collection

A number of different sources of evidence will be used, including interviews, documentation, archival records, and direct non-participant observations (see Table 2). This will allow us to address a broader range of behavioural, attitudinal, and historical issues [60]. Furthermore, by using a combination of methods and data, we will be able to validate and cross-check findings [60,82]. These approaches will provide methodological and data source triangulations [82-84,86].

**Table 2:** Sources of evidence

**Embedded unit of analysis**	**Sources of evidence**				
	**Interviews**				
	**Life history interviews** **(case studies)**	**Semi-structured interviews** **(key informants)**	**Documentation**	**Archival records**	**Direct non-participant observation**
**Field of family medicine**					
History and culture of family medicine		*	X		
In relation to the fields of medicine and power		*	X		
Capital at stake	*	*	X		
					
**Family physicians**					
Habitus	X				
Capital possessed	X		*	*	*
Individual and social contexts	X		*	*	*

X = primary source of evidence; * = source of evidence to corroborate and augment evidence from other sources.

Qualitative, in-depth interviews are one of the most important sources of case study information [60,84,86]. A life history interview approach will be employed as the primary source of data collection for the micro-level analysis (FPs). This approach has become very popular in social sciences, and professional studies [88]. According to Cándida Smith [89], "*Disjunction between discursive and pragmatic behaviour may be quite widespread and could provide insight into discrepancies in the political, economic, social, and cultural actions of social groups. The disjunction between subjective and objective factors in social relationships is an area for which oral history documents provide ideal sources of evidence*." Life history interviewing serves as an excellent means for understanding relationships, group interactions and memberships [90,91]. They can illuminate individual and collective actions, meanings, and modes of knowledge [89,91]. A life history narrative highlights the important influences, themes, issues, events, circumstances, feelings, and lessons of a lifetime [90,92]. It provides valuable perspective and understanding of the past and the present [90]. In the words of Atkinson [90], "*Telling a life story makes the implicit explicit, the hidden seen, the unformed formed, and the confusing clear*."

A minimum of 10 life history interviews (case studies) will be performed. A life history interview guide approach will be used. This approach involves predetermining a set of issues to be explored with each interviewee [82]. The life history interviews will mainly focus on the working life of the respondents. Interviewees will provide accounts of their reasoning and motivation for practicing family medicine, and details of their education and training. Interviewees will also provide detailed information about the following: family and key life events, professional trajectory, practice, field of family medicine (medical professional system, including social and professional networks), contexts (organizational, environmental and political), sources of information/scientific evidence, and economic evaluation in health care. Two interview sessions, of 90–120 minutes in length each, will be scheduled over a few days.

Documentation (study reports, administrative documents, or any other relevant documents) will play an important role in the data collection process [60,84,86]. In particular, documents will be the primary sources of evidence for the macro-level of analysis (field of family medicine). Documents will be provided by Departments of Family Medicine of the four Quebec Faculties of Medicine, professional associations, and the *Conseil québécois de développement continu professionnel des médecins *(CQDPCM; Quebec consultative coordinating organization for physicians' continuous professional development).

This historical document analysis will be enhanced by interviews of key individuals so as to acquire information that might not have become available through the documentation [84]. Key informants will be people who are particularly knowledgeable about the history of family medicine as a specialty in Canada and Quebec, and articulate about their knowledge. Key informants will also be particularly helpful in identifying both other relevant sources of evidence and key opinion leaders in Quebec's field of family medicine [60,82]. A open-ended, focused interview approach will be used. A focused interview, in which a respondent is interviewed for a short period of time, usually follows a certain set of questions [60,84,86]. An interview guide will only be created after the specific document analysis. Open-ended, semi-structured interviews, of 60–90 minutes in length each, will be undertaken with a minimum of one key informant from each of the following organizations (n = 9): Quebec College of Physicians (CMQ), College of Family Physicians of Canada (CFPC), Quebec College of Family Physicians (QCMF), Quebec Federation of General Practitioners (FMOQ), Departments of Family Medicine of the four Quebec Faculties of Medicine, and CQDPCM.

The use of various documents will also be used to corroborate and augment evidence from the life history interviews [60]. When relevant and available, various administrative documents and archival records will be used, including: organizational records of the clinic (type of organization, organization chart, organization design, history, culture, etc.), documents on the organization and availability of health and social services, at the local and regional territory levels (documents available on the web sites of the Ministry of Health and Social Services (MSSS), Health and Social Services Agencies (ASSS) and Health and Social Services Centres (CSSS)), documents on the population characteristics (sociodemographic and socioeconomic data, health status), at the local and regional territory levels (documents available on the web sites of the MSSS, ASSS and CSSS), survey data (such as census records), and other such records [60,84,86]. In particular, these sources of evidence will serve to check and enrich our understanding of the context (both individual and social) of the case and the capital (economic, cultural, social, symbolic) possessed [60,83].

Direct non-participant observation will serve as another source of evidence [60]. The observations will consist of casual data collection activities during each case study "site" visit [60,84,86]. Observational evidence (such as observations of environmental and organizational settings, health care resources available, relevant behaviours during the interview, and other relevant observations) will serve to check and enrich our understanding of the context of the case and the capital possessed [60,83].

Descriptive field notes will be written promptly after each observation and interview. These will be dated and include, among other things, quotations from the interviewee, the researcher's feelings, experiences and reactions (reflections, insights, ideas, inspirations, etc.), and field-generated insights and interpretations [82]

Interviews will be conducted according to the participant's or key informant's schedule and availability [84]. All interviews will be tape-recorded, with the written consent of the interviewees. All interviews and field notes will be transcribed into electronic format by the principal researcher, thoroughly immersing herself in the data [82]. This shall be completed within one week after each interview. If necessary, the interviewee shall be contacted for clarification [82]. The transcription of the interview will be sent to the interviewee for review.

Three principles will be followed to make the data collection process accessible and transparent for review. First, the data collected for each case study (such as field notes, documents, tabular materials, and narratives) will be compiled into a formal database [60,84,85]. Second, a chain of evidence shall be maintained so as to show clearly the links between the initial research questions asked, the data collected, and the ultimate case study conclusions [60,84]. Lastly, we will use a traditional paper logbook to track and record research activities (meeting notes, decisions, procedures, communications, calendar, telephone numbers, expenses, etc.).

#### Qualitative data analysis

Overall, it will be a two-step data analysis process. The macro-level of analysis will be conducted during the first months of the project. This first step will most probably generate hypotheses that will be tested in the second step, the micro-level of analysis.

The analytic induction approach to data analysis will be used [80,82]. In brief, this method involves formulating initial theoretical propositions (or hypotheses), examining the case study evidence, revising the propositions, and examining the evidence once again from a new perspective [60,80,82,84]. Therefore, data collection and analysis will be an iterative process, particularly at the beginning when the first interviews may help define new data collection and analysis needs, and adjust initial theoretical propositions [60,80,82].

We plan to use Miles and Huberman's [80] qualitative data analysis framework. These authors suggested helpful analytic techniques such as using arrays to display the data, creating displays (matrices and networks), tabulating the frequency of different events, ordering the information, using cross tabulations to examine the relationships between variables, and other such techniques to facilitate analysis [60,80,84,86]. The early step of data analysis will involve coding data, finding patterns, labelling themes, developing categories, and classifying [80,82]. A list of codes will be generated based on both the original framework and new themes introduced by the respondents. This means that codes will change and develop as fieldwork progresses [80]. This will produce a framework for organizing and describing the data collected during fieldwork [80,82].

We will conduct within-case and cross-case analyses of the data. For the within-case analysis, both variable-oriented and process-oriented (story-like) approaches will be combined for careful description and explanation.

For each case, we will initially develop a series of exploratory, descriptive within-case displays for drawing coherent conclusions about the phenomena in a bounded context. We will then develop a series of explanatory, causal within-case displays, with the purpose of explaining and predicting the phenomena. We will employ, as mentioned above, an analytic induction approach to build explanations [80]. For the cross-case analysis, we will use Miles and Huberman's [80] mixed strategy called 'stacking comparable cases', which combines both variable-oriented and case-oriented approaches for careful description and explanation. Once each case will be well understood, we will initially 'stacked' the case-level displays in meta-matrices, which will be further partitioned and clustered, permitting systematic comparison. Three types of exploratory, descriptive cross-case displays will be developed to deepen our understanding: conceptually-, case-, and time-ordered displays. We will then develop a series of explanatory, causal cross-case displays, with the purpose of explaining [80].

A computer-assisted qualitative analysis software (QSR NVivo 7) will be used. The software program facilitates the processes of data storage, data coding and categorizing, data retrieval, data comparing, and data linking [60,82].

### Ethical framework

For the macro-level analysis, the principal researcher (CL) will contact the Departments of Family Medicine of the four Quebec Faculties of Medicine, professional associations (CMQ, QCMF, CFPC, FMOQ), and CQDPCM to explain the objectives of the research project and invite them to participate. In particular, they will be invited to provide documents and suggest one individual within their organization who is particularly knowledgeable about the history of family medicine as a specialty in Canada and Quebec (key informant). Each key informant will receive an invitation letter explaining the objectives of the research project, along with a copy of the consent form.

For the micro-level analysis, each FP will receive an invitation letter explaining the objectives of the research project, along with a copy of the consent form. In both levels of analysis, the invitation letters will be signed by the principal researcher who has no relationship with the potential study participants. This procedure will prevent undue pressure to participate to the research project, and give adequate time to potential participants to consider their decision to volunteer.

Prior to giving consent, each participant will be fully informed by the researcher, who will conduct the interview, of the following: 1) background, purpose and objectives of the research; 2) research methodology; 3) foreseeable risks and potential benefits to the participant; 4) participant's participation is voluntary and that he/she has the right to withdraw from the study at any time; 5) how much time each participant is expected to give; 6) what use will be made of the information he/she provides; 7) participant's anonymity and confidentiality shall be fully protected; 8) data storage and protection; and 9) contact information.

In particular, each key informant will be informed that no financial compensation shall be received for their participation. However, each FP will be informed that he/she will receive a financial compensation of $150 (representing $75 per interview session). Each participant will be informed that their participation to the project does not entail any risk, and that it will contribute to a deeper understanding of the role of economic evaluation in the decision-making process of FPs as well as of the theory and practice of knowledge translation. Each participant will be informed that the final research report will be submitted as a thesis, and that manuscripts describing the results will be submitted for publication in scientific journals and for presentation at scientific meetings. He/she will be informed that no names will be re-transcribed and no explicit references to individual interviews will be made in these documents, and that results will be presented in an aggregated manner to avoid recognition of individuals, and settings. Strict measures will be taken to assure that only the research team has access to the research material, documents and databases. The participant will then be invited to sign two copies of the consent form before the interview begins. He/she will keep one copy for personal records, and give the other to the research team.

The research protocol, invitation letters and consent forms have received ethical approval from the *Comité d'éthique sur la recherche chez les êtres humains de la Faculté de médecine *(CERFM; Ethics Committee for Human Research of the Faculty of Medicine) of the University of Montreal on July 10, 2007 (Reference Number: CERFM-84 (07) 4#246).

## Discussion

To date, there is little evidence on the role of economic evaluation in the health care decision-making process taking place at the micro level. It is at that particular level that most decision-making occurs, and thus, where economic evaluation evidence should have the most extensive influence. First, our research could contribute to a deeper understanding of the social processes that influence FPs' schemes of perception, thought, appreciation and action with respect to the role of economic evaluation evidence in clinical decision-making, and the potential influence of economic evaluation evidence on everyday clinical decision-making. It could also contribute to a deeper understanding of the FPs' willingness to contribute to efficient, fair and legitimate resource allocation. These are essential first steps in making suggestions for improvements in the future. Second, our research could contribute to an increased role and applicability of economic evaluation on clinical decision-making, for example by enhancing methods and approaches, report presentation and diffusion of economic evaluations, or by proposing interventions centred on physicians which would enhance the perception, attitudes, understanding and influence of economic evaluation evidence by these decision-makers.

One of the most important challenges facing the world of research today is to develop effective knowledge translation strategies specific to the primary care sector [21]. It is also a highly important issue for health care systems in Canada, Quebec and elsewhere in the world. Our research could contribute to a deeper understanding of the theory and practice of knowledge translation, and the development of a conceptual model specific to the micro level of decision-making highlighting complex relationships between various agents and structures, and economic evaluations. This could allow economic evaluation researchers to develop effective knowledge translation strategies specific to the primary care sector. Lastly, these potential theoretical outcomes of our research will provide valuable lessons for the research and scientific community, particularly for those who are active in heath technology assessment (HTA), health economics and pharmacoeconomics, health policy, health services, and health care organization research.

The final research report will be submitted as a thesis. In particular, a minimum of three manuscripts will be submitted for publication in peer-reviewed scientific journals. Presentations at provincial, national and international scientific meetings will be made. Lastly, the involvement of provincial and national professional organizations in the research process will foster an effective two-way knowledge transfer between the researchers and the organizations. Research results will be presented to representatives of the four Quebec Faculties of Medicine, professional associations (CMQ, QCMF, CFPC, FMOQ), CQDPCM and other interested organizations (provincial and national HTA Agencies, etc).

## Competing interests

The authors declare that they have no competing interests.

## Authors' contributions

CL is the principal and coordinating investigator for the research study. She was responsible for the conception, design and methods of the study, obtained research funding, wrote the draft manuscripts, responded to authors' comments and completed the final manuscript. APC and MDB provided supervision and guidance, contributed to the conception, design and methods of the study and critically commented on draft manuscripts. All authors read and approved the final manuscript.

## Pre-publication history

The pre-publication history for this paper can be accessed here:


